# Supplementary material for: Fungi with history: Unveiling the mycobiota of historic documents of Costa Rica
Source: PLoS One. 2023 Jan 18;18(1):e0279914. doi: 10.1371/journal.pone.0279914 (PMC9847896; doi:10.1371/journal.pone.0279914)

Figure S1. Multispectral photograph of page 127 of the Independence Act. **A**. Reflectance ultraviolet photograph. **B**. Fluorescence ultraviolet photograph. **C**. Visible photograph. Surface indicate an oxidation process from the cellulose and iron, probably caused by both abiotic and biotic factors.

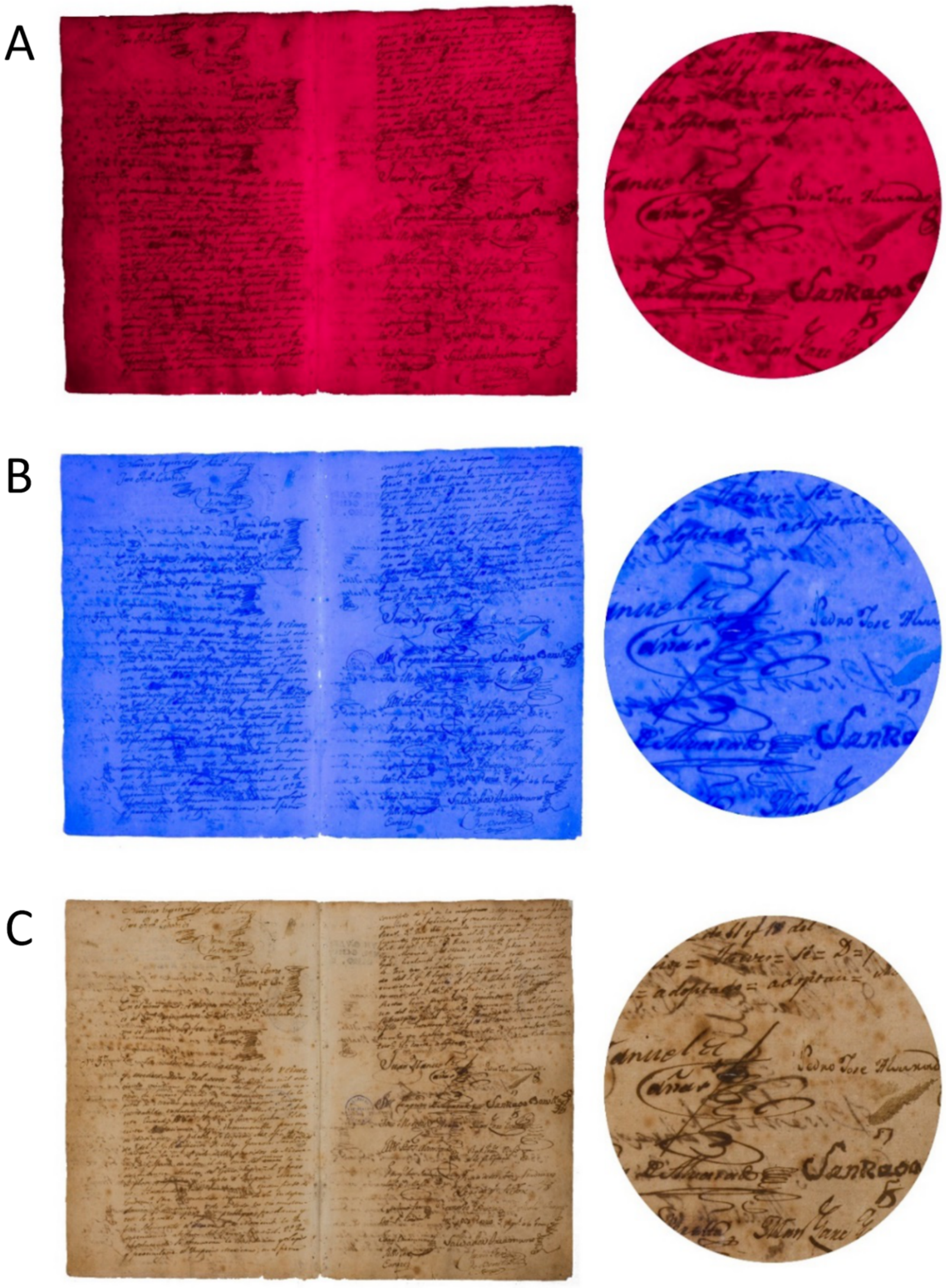

Supplement: S1 Fig — A. Reflectance ultraviolet photograph. B. Fluorescence ultraviolet photograph. C. Visible photograph. Surface indicate an oxidation process from the cellulose and iron, probably caused by both abiotic and biotic factors. (PDF) [file pone.0279914.s001.pdf]
