# Supplementary material for: Fungi with history: Unveiling the mycobiota of historic documents of Costa Rica
Source: PLoS One. 2023 Jan 18;18(1):e0279914. doi: 10.1371/journal.pone.0279914 (PMC9847896; doi:10.1371/journal.pone.0279914)

Figure S2. Attenuated total reflectance Fourier transform infrared spectra (ATR-FTIR) of the (**A**) Independence Act; (**B**) Political Constitution, 1949 (1991 replica); (**C**) Cloudy Days Act (folio 2); (**D**) Cloudy Days Act (folio 1); (**E**) Guatemalan Series 1539; and (**F**) Guatemalan Series 1549. Also see supplementary Table S1.

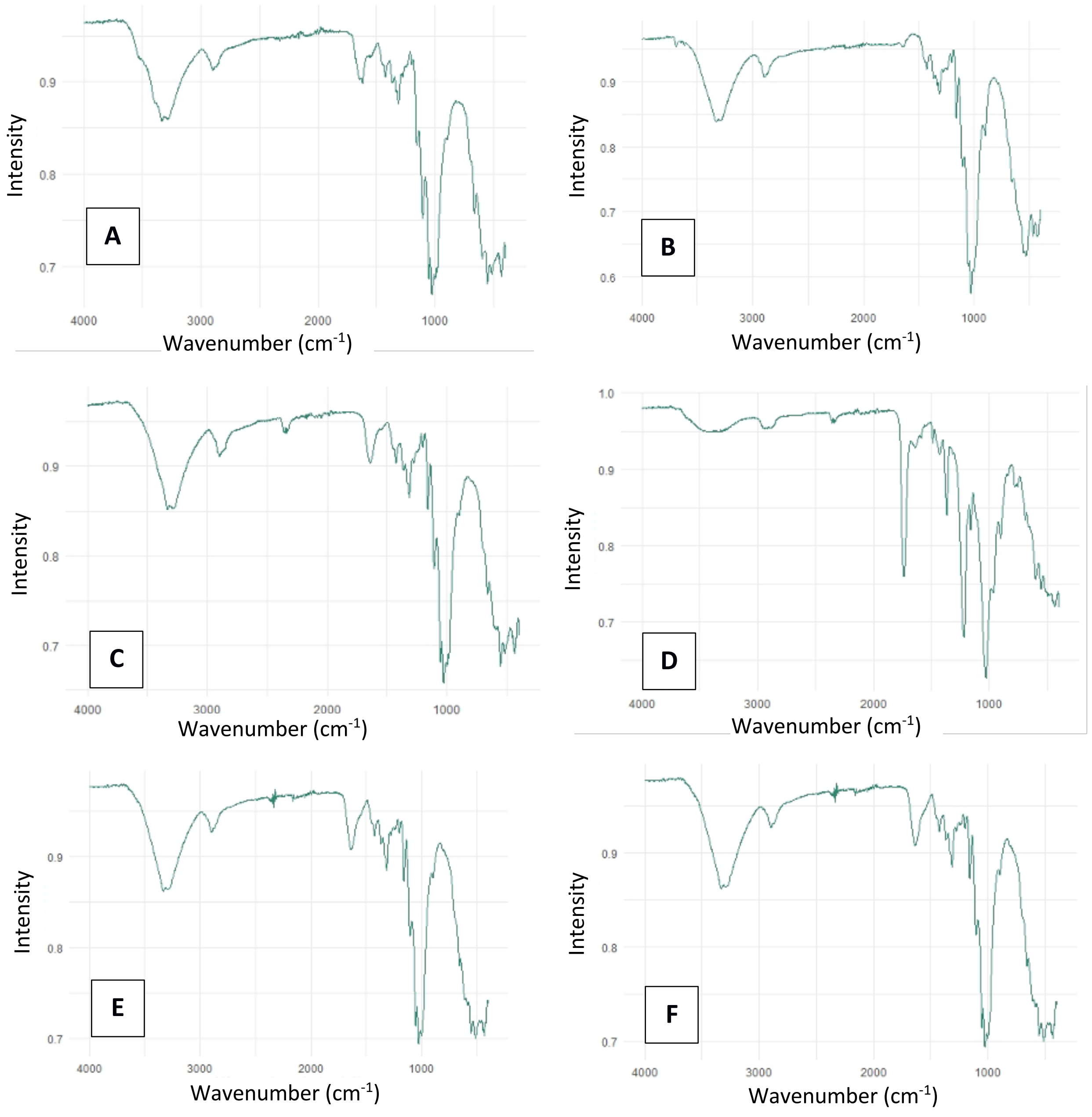

Supplement: S2 Fig — Attenuated total reflectance Fourier transform infrared spectra (ATR-FTIR) of the (A) Independence Act; (B) Political Constitution, 1949 (1991 replica); (C) Cloudy Days Act (folio 2); (D) Cloudy Days Act (folio 1); (E) Guatemalan Series 1539; and (F) Guatemalan Series 1549. Also see S1 Table. (PDF) [file pone.0279914.s002.pdf]
