# Supplementary material for: Fungi with history: Unveiling the mycobiota of historic documents of Costa Rica
Source: PLoS One. 2023 Jan 18;18(1):e0279914. doi: 10.1371/journal.pone.0279914 (PMC9847896; doi:10.1371/journal.pone.0279914)

Figure S3. Fungal isolates recovered from the historical documents from the NACR. **A.** 1539-A1P, *Purpureocillium lilacinum* **B.** 1549-1A1P, *Penicillium compactum*. **C.** 1549-4A1C, Herpotrichiellaceae. **D.** AI1-A1C, *Cladosporium* sp. **E.** AI3-A1P, *Aspergillus hiratsukae* **F.** CP1-A1C, *Periconia* sp. **G.** CP1-A1P, *Cladosporium* sp. **H.** CP1-A2C, *Pestalotiopsis microspora* **I.** CP1-A2P, *Cladosporium* sp. **J.** CP1-A3C, *Trametes hirsuta* **K.** CP1-A3P, *Unidentified Psathyrellaceae*. **L.** CP2-A1C, Unidentified Pleosporales. **M.** CP2-A1P, *Purpureocillium lilacinum* **N.** CP2-A2C, *Pestalotiopsis trachycarpicola* **O.** CP2-A2P, *Coprinellus* sp. **P.** CP2-A3C, *Acremonium persicinum* **Q.** CP2-A3P, *Beauveria bassiana* **R.** CP2-A4C, *Acremonium persicinum* **S.** CP2-A4P, *Cyphellophora* aff. *pluriseptata*. **T.** CP2-A5C, *Penicillium sumatraense* **U.** ND1-A1P, *Penicillium steckii* **V.** ND2-A1P, *Cladosporium* sp.

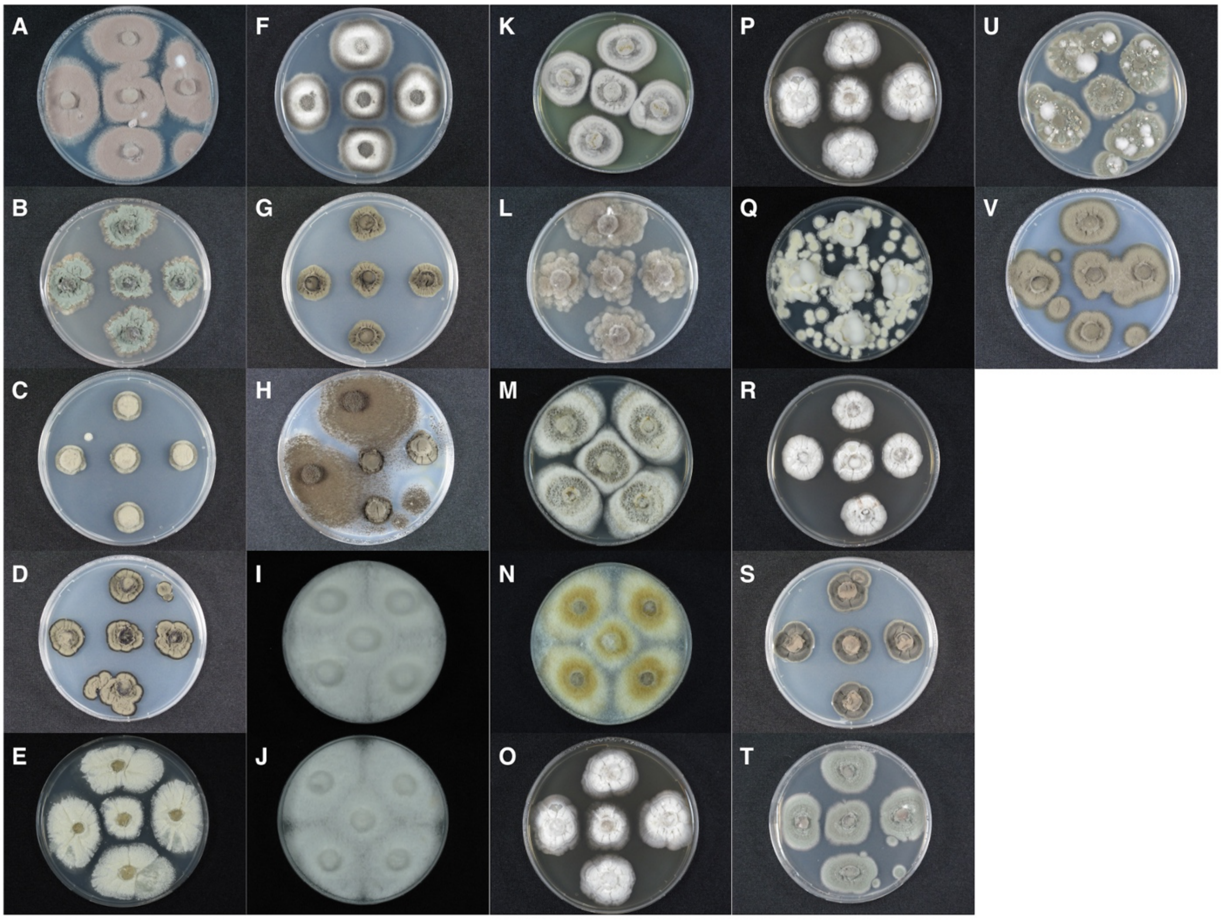

Supplement: S3 Fig — A. 1539-A1P, Purpureocillium lilacinum B. 1549-1A1P, Penicillium compactum. C. 1549-4A1C, Herpotrichiellaceae. D. AI1-A1C, Cladosporium sp. E. AI3-A1P, Aspergillus hiratsukae F. CP1-A1C, Periconia sp. G. CP1-A1P, Cladosporium sp. H. CP1-A2C, Pestalotiopsis microspora I. CP1-A2P, Cladosporium sp. J. CP1-A3C, Trametes hirsuta K. CP1-A3P, Unidentified Psathyrellaceae. L. CP2-A1C, Unidentified Pleosporales. M. CP2-A1P, Purpureocillium lilacinum N. CP2-A2C, Pestalotiopsis trachycarpicola O. CP2-A2P, Coprinellus sp. P. CP2-A3C, Acremonium persicinum Q. CP2-A3P, Beauveria bassiana R. CP2-A4C, Acremonium persicinum S. CP2-A4P, Cyphellophora aff. pluriseptata. T. CP2-A5C, Penicillium sumatraense U. ND1-A1P, Penicillium steckii V. ND2-A1P, Cladosporium sp. (PDF) [file pone.0279914.s003.pdf]
