# Supplementary material for: Fungi with history: Unveiling the mycobiota of historic documents of Costa Rica
Source: PLoS One. 2023 Jan 18;18(1):e0279914. doi: 10.1371/journal.pone.0279914 (PMC9847896; doi:10.1371/journal.pone.0279914)
Supplement: S1 Table — (PDF) [file pone.0279914.s004.pdf]

**Table S1**. Infrared band assignments for cotton-based papers, according to Hajji et al. [22].

| Band Position ($cm^{-1}$) | Assignment* |
|---|---|
| **3338, 3284** | ν(OH): Stretching of H attached to OH group |
| **2900** | ν(CH): stretching of CH groups |
| **1652** | δ(OH): bending mode of adsorbed water |
| **1429** | δs(CH2): symmetric bending of CH2 at C6 |
| **1369** | 1369 δ(CH): In-plane CH bending vibrations |
| **1334** | 1334 δ(OH): In-plane bending of OH in crystalline cellulose |
| **1315** | 1315 γ(CH2): CH wagging at C6 of amorphous cellulose |
| **1203** | 1203 δ(C-O-H): In-plane bending in C6 |
| **1161** | 1161 ν(C-O-C): Tensile vibration of the β-glucosidic bond in crystalline cellulose |
| **1106** | νa (glucose ring): in-plane tensile vibration of glucose ring |
| **1053** | ν(C-O): stress vibration in C3 |
| **1018** | ν(C-O), ν(C-C): stretching of C-O and C-C at C6 |
| **663** | δ(C-OH): out-of-plane bending |

*ν: stretching, δ: bending, γ: wagging, a: asymmetrical, s: asymmetrical
