# Supplementary material for: Fungi with history: Unveiling the mycobiota of historic documents of Costa Rica
Source: PLoS One. 2023 Jan 18;18(1):e0279914. doi: 10.1371/journal.pone.0279914 (PMC9847896; doi:10.1371/journal.pone.0279914)
Supplement: S2 Table — Measurements shown are average ± standard deviation, and minimum and maximum in parentheses. (PDF) [file pone.0279914.s005.pdf]

Table S2. Percentage of iron and calcium in the inks of the Independence Act (AI), Cloudy Days Act folio 1 (N1), Cloudy Days Act folio 2 (N2). Measurements shown are average ± standard deviation, and minimum and maximum in parentheses.

Sample	n	% Fe mass	% Ca mass
**AI**	19	(0.2–)1.4±1.2(–4.5)	(0.5–)0.7±0.2(–1.3)
**N1**	17	(0.1–)1.1±1.0(–3.2)	(0.6–)1.4±0.5(–2.3)
**N2**	16	(0.1–)2.0±2.6(–7.5)	(0.7–)1.5±0.5 (–2.2)

n: number of measurements.
